# Complete Mitochondrial Genomes of *Pluvialis fulva* and *Charadrius dubius* with Phylogenetic Analysis of Charadriiformes

**DOI:** 10.3390/genes15121642

**Published:** 2024-12-21

**Authors:** Kuo Sun, Qingxiong Wang, Kun Bian, Feiran Li, Jie Tang, Lijuan Suo, Xiang Hou, Chao Yang

**Affiliations:** 1 Shaanxi Key Laboratory of Qinling Ecological Security, Shaanxi Institute of Zoology, Xi’an 710032, China; sunkuo@snnu.edu.cn (K.S.); wqx546@163.com (Q.W.); biankun@ms.xab.ac.cn (K.B.); i_am_lfr_ttm@163.com (F.L.); yaya184@xab.ac.cn (J.T.); suolijuan@xab.ac.cn (L.S.); hx426108@163.com (X.H.); 2 Shaanxi Provincial Field Observation & Research Station for Golden Monkey, Giant Panda and Biodiversity, Xi’an 723400, China; 3 Baoji City Forest Musk Engineering Technology Research Center, Feng County, Baoji 721700, China

**Keywords:** Charadriidae, intraspecies, mitogenomic phylogeny, *Charadrius dubius*, *Pluvialis fulva*

## Abstract

Background: Plovers (Charadriidae), within the order of Charadriiformes, a group of modern birds distributed worldwide, are a frequent subject of molecular phylogenetic studies. While research on mitochondrial genome (mitogenome) variation within the family Charadriidae, especially intraspecific variation, is limited. Additionally, the monophyly of *Charadrius* and the phylogenetic placement of *Pluvialis* remain contentious. Nevertheless, recent studies utilizing complete mitogenomes from available databases to construct phylogenetic trees for Charadriidae and Charadriiformes remain scarce. Methods: This study aims to explore mitogenome variation within *Charadrius dubius* and clarify the phylogenetic placement of *Pluvialis fulva.* We sequenced the complete mitogenome of six *C. dubius* and one *P. fulva*, and all additional available mitogenomes were integrated within Charadriiformes. The average complete mitogenome length of *C. dubius* is 16,889 bp, and *P. fulva* is 16,859 bp. Results: Our results support the suggestion that the monophyly of *Charadrius* and *P. fulva* is nested within Charadriidae. The phylogenetic analysis of Charadriiformes based on mitogenomes strongly supports the recognition of three major shorebird clades: Charadrii, Lari and Scolopaci, with Lari and Scolopaci identified as sister clades. Conclusions: Our study reinforces the credibility of the inferred evolutionary relationships within Charadriidae and Charadriiformes.

## 1. Introduction

Shorebirds (Aves: Charadriiformes) are widely distributed globally—current taxonomic arrangements recognize 19 families and 386 species [1,2]. Within Charadriiformes, plovers (Charadriidae) represent a diverse and widespread group of birds, encompassing 71 species distributed across 12 genera. This diversity and broad ecological and behavioral adaptations make Charadriidae an intriguing subject for studying shorebird evolution [3]. Among Charadriidae, the genus *Charadrius* includes approximately 40 species of plovers, a diverse group of shorebirds, but the monophyly of *Charadrius* remains controversial. The genus *Pluvialis* includes four migratory species; despite their wide distribution, the phylogenetic relationships within this genus remain unresolved [2,4,5]. Specifically, there are three prominent hypotheses about the phylogenetic position of the genus *Pluvialis*: (i) it is sister to Charadriidae [6,7]; (ii) it is sister to Haematopidae, Recurvirostridae and Ibidorhynchidae [8,9]; and (iii) it is sister to Charadriidae, Haematopidae, Recurvirostridae and Ibidorhynchidae [10]. A recent supermatrix analysis supported the first hypothesis [2].

Mitochondrial genomes (mitogenomes) have a high evolutionary rate and maternal inheritance characteristics, making them widely used in molecular evolution and phylogenetics, especially for closely related species [11,12]. The number of complete mitogenomes has been progressively rising due to advancements in sequencing technology [13]. These genomes, with their intra- and interspecific variation, serve as valuable markers for investigating animal evolution, including humans, particularly in fields such as taxonomy, systematics, ecology and population biology [14,15]. The completed mitogenome has been determined for 183 unique sequences of 103 species in Charadriiformes. Within Charadriidae, 37 completed mitogenome records of 13 species are available in NCBI [2]. Nevertheless, intraspecific variation in plovers’ mitogenomes is rarely studied, and *Pluvialis’* phylogenetic position remains unclear, along with the lack of a comprehensive phylogenetic analysis of Charadriiformes based on complete mitogenomes, which remains underexplored in previous studies [2,6,9,16].

Here, we sequenced and assembled complete mitogenomes of two species (*Pluvialis fulva*, *Charadrius dubius*) from Charadriidae sampled in China. The little ringed plover, *C. dubius*, is widely distributed from Africa to Eurasia, with breeding grounds spanning Europe and India to East Asia. It is characterized by a black mask around its face with yellow eye rings [17]. The Pacific golden plover, *P. fulva,* is also a strongly migratory species [18], extending its range south to Asia, Australasia and the Pacific islands [19,20]. To investigate the intraspecific variation in *C. dubius*, we compared mitogenomes from multiple individuals. Additionally, we sequenced a new mitogenome of *P. fulva* to clarify its phylogenetic position within Charadriidae further. To ensure the robustness of the phylogenetic tree of Charadriidae and address the lack of a comprehensive phylogenetic analysis of Charadriiformes based on complete mitogenomes, we constructed a phylogenetic tree using all available complete mitogenomes of Charadriiformes from GenBank (Until 26 June 2024) [2,6]. Our study provides a solid framework for understanding evolutionary dynamics and relationships of plovers and shorebirds.

## 2. Materials and Methods

### 2.1. Specimen Collection and DNA Extraction

This study collected specimens from natural populations, with pectoral muscle samples preserved in 95% ethanol (Table 1). Total genomic DNA was extracted from the muscle tissue using the Rapid Animal Genomic DNA Isolation Kit (Sangon Biotech Co., Ltd., Shanghai, China), following the manufacturer’s instructions. The mitogenomes were assembled using low-coverage next-generation sequencing. We collected all available sequences of Charadriiformes. It should be noted that among the sequences we collected, five sequences were deleted in the phylogenetic analysis. The specific accession numbers and reasons for removing these sequences are stated in Table S1. A total of 114 sequences from 86 species were included in the phylogenetic analysis (Table S1).

### 2.2. Library Preparation and Sequencing

Biomarker Technologies (Biomarker Technologies Co., Ltd., Beijing, China) performed DNA library construction and sequencing. On the Illumina HiSeq 4000 platform (Illumina, San Diego, CA, USA), 350 bp paired-end libraries with 150 bp read length were constructed and sequenced. Each sample generated an average of 2 Gb of raw data. Sequencing reads from seven individuals were cleaned by removing adapter sequences and low-quality reads. 

### 2.3. Mitogenome Assembly and Annotation

Geneious Prime (version 2022.1.1) was used to manually assemble each mitogenome using the overlap–layout–consensus approach [21], which consists of firstly identifying overlaps (O) between reads, secondly constructing a layout (L) based on overlaps and, finally, deriving the consensus (C) sequence from the layout. The process involved several iterations of mapping with suitable mismatch rates. The initial mapping was performed against a reference mitogenome, while subsequent mappings were conducted using the progressively lengthening mitochondrial sequence. The gene-coding sequences, tRNAs and rRNAs were annotated using Mitos2 [22], and the reference data selected were metazoa, with the vertebrate mitochondrial genetic code specified. The annotations were manually examined in Geneious Prime (version 2022.1.1) [21]. Proksee was used to generate the mitogenome map (https://irscope.shinyapps.io/Chloroplot/, accessed on 23 May 2024). The mitogenome sequence of grey plover *Pluvialis squatarola* (accession number: MT561267) was used as a reference [23].

### 2.4. Comparative Mitogenomic Analyses

Based on the following formulas, we estimated compositional skewness levels: AT skew = (A − T)/(A + T) and GC skew = (G − C)/(G + C). PhyloSuite was used to calculate nucleotide composition and relative synonymous codon usage (RSCU) [24]. Based on the following formulas, we estimated compositional skewness levels: AT skew = (A − T)/(A + T) and GC skew = (G − C)/(G + C). Ka and Ks rates and nucleotide diversity (Pi) for each PCG within Charadriidae were calculated using the kaks function from the R package seqinr [25] and the nuc.div function from the R package pegas [26], respectively.

### 2.5. Mitogenomic Phylogenetic Analyses

We first searched and downloaded all available mitogenome sequences of Charadriiformes from NCBI. In total, 114 sequences were included in the phylogenetic analyses, comprising 118 complete mitochondrial sequences from Charadriiformes, representing 45 genera and 13 families, and an outgroup sequence from *Balearica regulorum* of the Gruiformes. Among these sequences, 107 were obtained from NCBI, and 7 were generated in this study. Based on a genome-scale analyses [27], which identified Gruiformes as the sister group to shorebirds, *B. regulorum* from the Gruiformes was assigned as the outgroup, following the methodology described by Černý and Natale [2]. A detailed description of the samples used in the phylogenetic analysis is provided in Table S1.

Geneious Prime (version 2022.1.1) was used to extract the sequences of 13 mitochondrial protein-coding genes (CDSs) from all 114 mitogenomes [21]. For each gene, transitional alignment was performed using default parameters in MUSCLE [28]. Concatenated alignments were partitioned by gene and codon position, and the best substitution model was selected using ModelFinder [29] in IQ-TREE v2.0.622 (option -m MFP + MERGE). Node support values were calculated using an ultrafast bootstrap approximation [30] with 1000 replicates (option -B 1000). A total of 13 PCGs were partitioned using PartitionFinder2 (Lanfear et al., 2016), offering partitions by gene and codon. We used MrBayes v3.2.7 [31] to perform the Bayesian inference (BI) analysis, implementing two independent runs with four concurrent Markov chains for 10,000,000 generations, with sampling every 1000 generations. The convergence and mixing of the chains of each analysis were evaluated using Tracer v1.7.1 [32] to check that the ESS values were all superior to 200. Phylogenetic trees were visualized and annotated using ggtree v3.13.0 [33].

## 3. Results and Discussion

### 3.1. C. dubius and P. fulva Mitogenome Structure and Organization

**Figure 1:**
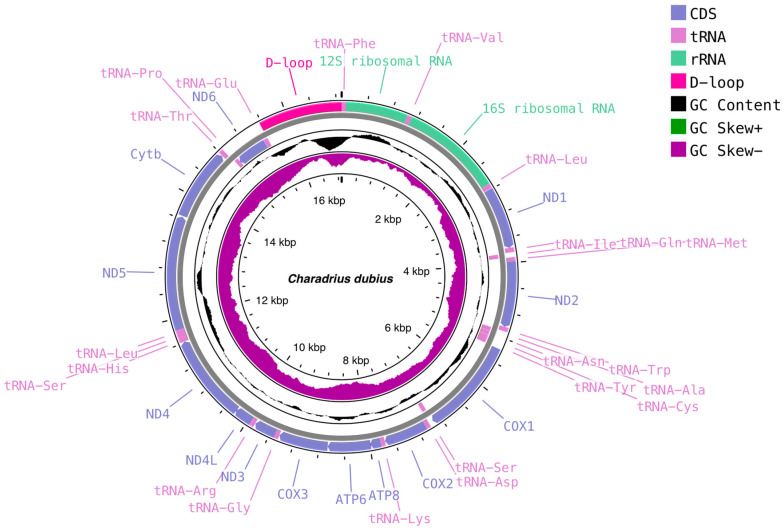

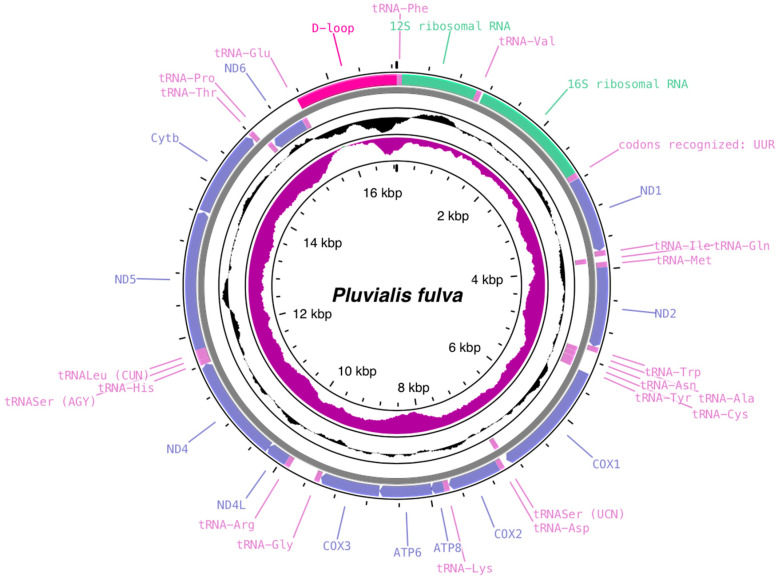
*Charadrius dubius* and *Pluvialis fulva* mitogenomes. The genes in the outermost circle are transcribed clockwise, and the genes in the inner circle are transcribed counterclockwise. GC skews and G + C content are shown in the inside circles.

We assembled seven mitogenomes of two species for the Charadriidae, including six *C. dubius* and one *P. fulva* (Figure 1; Table 1 and Table 2). All seven mitogenomes were complete. Six specimens *of C. dubius* and one specimen of *P. fulva* sequenced in this study all have circular double-stranded DNA molecules, each comprising 13 protein-coding genes (PCGs), two ribosomal RNAs (*rRNAs*), 22 transfer RNAs (*tRNAs*) and a noncoding D-loop region (Figure 1). A total of 12 of the 13 PCGs and 14 of the 22 tRNAs were encoded by the J-strand. The remaining eight genes were encoded by the minority strand (N-strand) (Figure 1). All seven mitogenomes in this study had a D-loop region located between tRNA (Glu) and tRNA (Phe) (Figure 1), which was consistent with most bird mitogenomes [34].

The length of *C. dubius* mitogenomes in this study ranged from 16,883 bp (No. 40) to 16,926 bp (No. 146), similar to *C. dubius* sequenced in a previous study (16,864 bp, Table 2) [35]. The mitochondrial DNA sequences (without D-loop region) of the six *C. dubius* samples are 15,538, 15,539, 15,522, 15,517, 15,497 and 15,494 bp in size, respectively. The gene arrangements of these six *Charadrius* mitogenomes were very conserved and highly consistent with the previously sequenced *C. dubius* (Figure 1). We did not find mitochondrial gene rearrangements in *C. dubius*, which is consistent with most investigations in birds [36]. The overall GC content was similar for the six mitogenomes of *C. dubius* with an average of 44.6% (Table 2). No significant differences in GC content were found between protein-coding genes, tRNA and rRNA. We found substantial differences in the GC content of rRNA in the mitogenomes of *C. dubius* individuals from different locations. For example, the GC content for individuals with protein-coding genes, *rRNA* genes and *tRNA* genes was 45.6%, 45.7% and 45.5%, respectively. These slight variations may reflect selective pressures from different environments, potentially affecting genome stability and function [36,37]. Additionally, variations in GC content could result from differential mutation rates and selection pressures [36]. Identifying these differences provided a deeper understanding into mitogenome adaptive and evolutionary dynamics.

The total length of the mitogenome of *P. fulva* was 16,859 bp. Excluding the D-loop region, the length was 15,541 bp, similar to the length of 15,538 bp reported in a previous study [6]. The gene arrangement in the mitogenome of *P. fulva* was extremely conserved and consistent with that of a previously reported mitogenome of this species (NC_033966) [18].

### 3.2. Codon Usage

*C. dubius* and *P. fulva* had very similar start and stop codons for the 13 protein-coding genes (PCGs). Only the stop codon of the *ND5* gene differed: TAA in the former and TAG in the latter (Table S2). In *C. dubius*, *ATP8* was the most minor PCG, whereas *ND5* was the most prominent. The length of *ND5* in *C. dubius* and *P. fulva* is 1815 bp (Table S2). ATG is the typical start codon for PCGs in *C. dubius* and *P. fulva*, whereas ATC was typical for *ND3* (Table S2), while GTG was typical for *ND5* and *COX1*. Columbidae and Phasianidae [38] have also been observed to have the unusual GTG start codon in *COX1*.

There was a great deal of variation in the stop codons of the 13 PCGs in *C. dubius* and *P. fulva*. The stop codons of *COX2*, *ATP8*, *ATP6*, *ND3*, *ND4L*, *ND5* and *CYTB* were TAA, while *ND2* and *ND6* terminated with TAG. *COX1*, *ND1* and *ND5* terminated with AGG or ATC, while *COX3* and *ND4* terminated with the truncated stop codon. It was common in metazoan mitogenomes [39] to have incomplete stop codons, such as TA or T, which could be converted to TAA during mRNA maturation [40].

The lengths of each of the 13 PCGs in *C. dubius* and *P. fulva* were nearly identical. However, the length of the ND6 gene in *C. dubius* varied significantly, with lengths of 528 and 546 bp, respectively. In contrast, the *ND6* gene in *P. fulva* had a length of 519 bp (Table S2).

**Figure 2:**
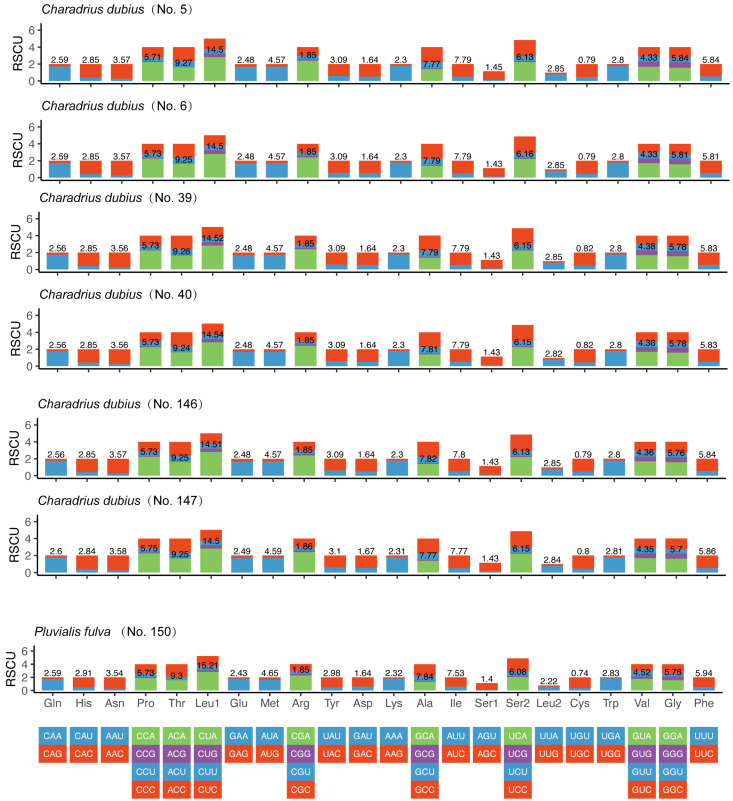
A comparative analysis of the mitogenomes of *Charadrius dubius* and *Pluvialis fulva* in terms of RSCU.

In Figure 2, we presented codon usage, relative synonymous codon usage (RSCU) and codon family proportions (corresponding to amino acid usage) for two shorebird species (*C. dubius* and *P. fulva*). The RSCU was statistically analyzed and we found that in *C. dubius*, the six species had similar codon usage frequencies overall. Leucine1 (14.5–14.54%), Thr (9.24–9.27%) and Ala (7.77–7.82%) were the most abundant amino acids in the PCGs of *C. dubius*; in contrast, cys (0.79–0.82%) and asp (1.64–1.67%) were relatively scarce (Figure 2). A similar situation was seen in *P. fulva* (Leu, 17.43%; Thr, 9.3%; Ala, 7.85%; Figure 2).

**Figure 3:**
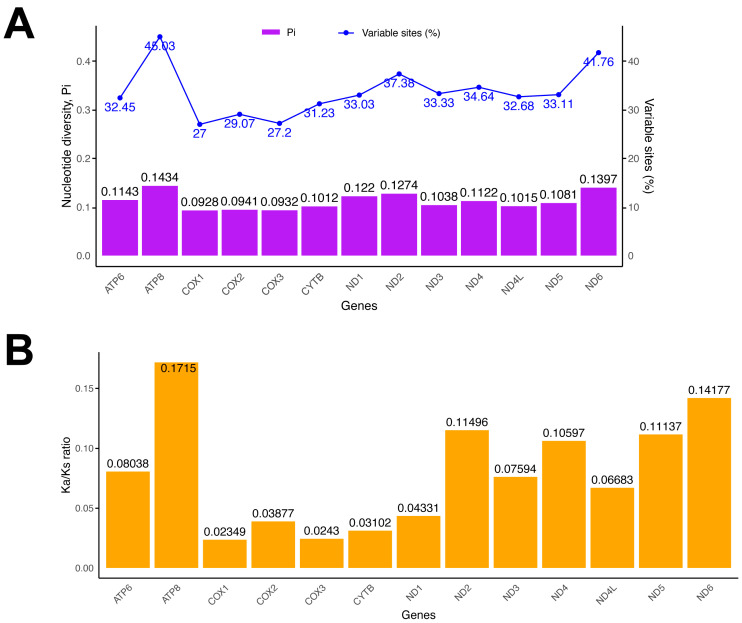
Evolutionary rates of mitochondrial genes for 13 species from 20 Charadriidae sequences. (**A**) The diversity of nucleotides and the percentage of variable sites. (**B**) The ratio of non-synonymous substitutions to synonymous substitutions.

Nucleotide diversity varied significantly across various mitochondrial genes (Figure 3). For *COX1* and *ATP8*, the average nucleotide diversity was 0.0928 and 0.1434, respectively, with the proportion of variable DNA sites spanning from 27% in *COX1* to 45.03% in *ATP8* (Figure 3A). *COX1* was a slow-evolving gene, while *ATP8* evolves rapidly. To gain a deeper understanding of the selective pressure on mitochondrial PCGs among Charadriidae, the average Ka/Ks ratio for each PCG was calculated and compared (Figure 3B). A Ka/Ks ratio of 1 indicates neutral mutations, a Ka/Ks ratio of less than 1 indicates negative selection, and a Ka/Ks ratio of greater than 1 indicates positive selection [41,42]. For all PCGs in Charadriidae mitogenomes, the Ka/Ks ratios were consistently lower than 1, suggesting purifying selection affected all PCGs. There was a high rate of substitution for *ATP8* (0.17), while a low rate of evolution for *COX1* (0.023) (Figure 3B).

Similarly to most other birds [43,44], all 10 Charadriidae birds had lower G + C contents than A + T contents (Table 3). The analysis of nucleotide composition and skew in the mitogenomes of the 10 Charadriidae species revealed significant variability among the different species (Table 3). AT skew values averaged 0.13 to 0.15, while GC skew values ranged from −0.40 to −0.38. In addition, *C. dubius* demonstrated consistent AT skews of 0.14 across multiple samples, with GC skews ranging between −0.38 and −0.39, reflecting slight variations in AT and GC content among the samples.

### 3.3. Phylogenetic Analyses

Our study was the most comprehensive phylogenetic analysis of Charadriidae based solely on mitochondrial data, utilizing 13 mitochondrial genes, including mitogenomes of 11 species from Charadriidae individuals belonging to three genera. We constructed phylogenetic trees of 114 Charadriiformes species using both ML and BI methods and found that the topologies obtained from both methods were identical. This consistency between ML and BI approaches suggests a robust dataset and a reliable phylogenetic structure, reinforcing the credibility of the inferred evolutionary relationships within Charadriiformes. Additionally, our results further support taxonomic recognition of a clade formed by the other members of Lari (i.e., Lari excluding Turnicidae), which has been designated as Larida [2]. *Hesperoburhinus* (Burhinidae), *Chionis* (Chionidae) and *Pluvianellus* (Pluvianellidae) formed a well-supported clade that is sister to all other Charadrii members, referred to as Chionida by Černý and Natale [2]. Rostratulidae and Jacanidae formed a clade with strong support and were sisters to Scolopacidae, forming a clade named Jacanida [2]. The rare Saunders’ Gull *Chroicocephalus saundersi* was represented by two mitogenome sequences and was sister to a clade formed by *Chroicocephalus*, *Ichthyaetus* and *Larus*. The non-monophyly of *Chroicocephalus* and the isolated position of Saunders’ Gull support the recognition of a monotypic genus: *Saundersilarus* [45].

**Figure 4:**
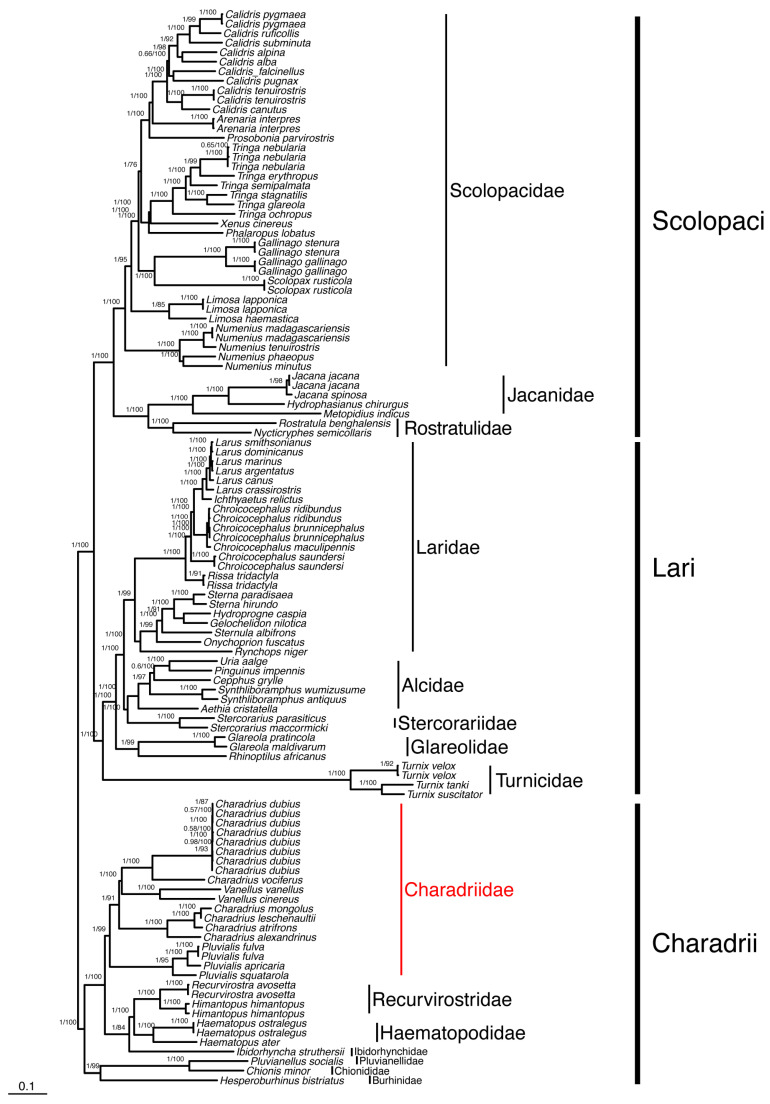
The BI and ML phylogenetic tree of 114 Charadriiformes species based on 13 mitochondrial CDS. The numbers above branches indicate posterior probability (BI) and bootstrap value (ML), respectively. Families and suborders were labeled on the right. Accession numbers and superfamily labels are shown in Figure S1.

**Figure 5:**
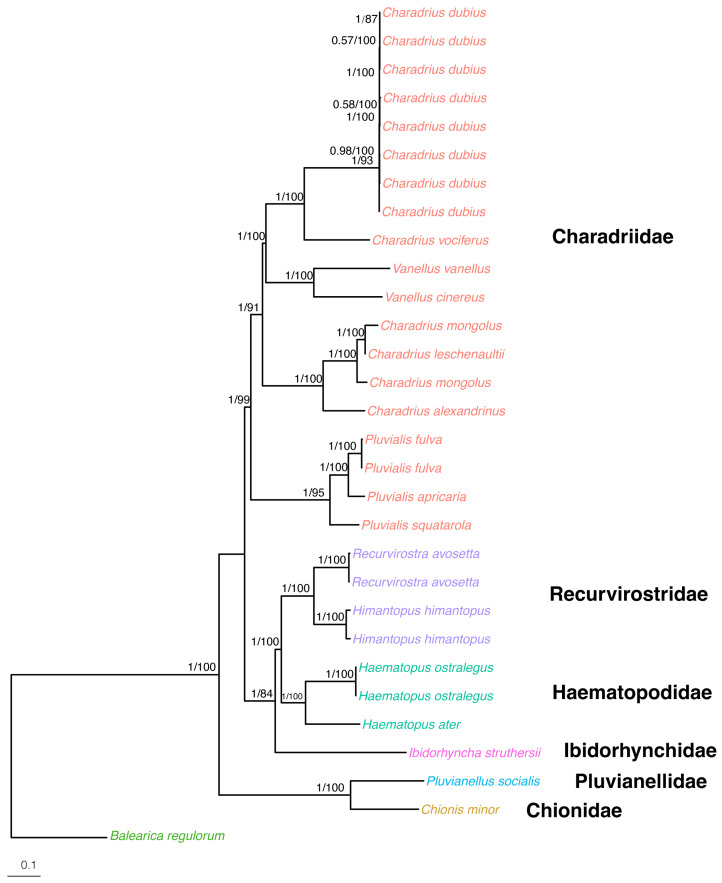
The BI and ML phylogenetic tree constructed for suborder Charadrii using 13 mitochondrial protein sequences. It was first built with 86 species of 114 mitochondrial sequences of Charadriiformes and later pruned down to 19 species of 31 sequences with Charadrii. In total, there are six different families (shown with different colors) and *Balearica regulorum* is included as an outgroup (Gruiformes). The numbers above branches indicate posterior probability (BI) and bootstrap value (ML), respectively. Families were labeled on the right.

Our results supported a close and strongly supported relationship between *Pluvialis* and Charadriidae (Figure 4 and Figure 5). The result suggested that Charadriidae was monophyly and supported by high bootstrap values and posterior probability (Figure 4 and Figure 5). *Charadrius* was monophyletic, although it appeared to be polyphyletic, while *C. dubius* and *C. vociferus* were found to be more closely related to two species of *Vanellus* than to other *Charadrius* species, which has also been found in other studies [2,4,5]. The non-monophyly of the genus *Charadrius* results from incorrect taxonomic delimitation of the genus. Specifically, *Charadrius morinellus* is now named *Eudromias morinellus*, *C. mongolus* and *C. leschenaultii* are now placed in the genus *Eupoda*, and *C. alexandrrinus* was moved to the genus *Ochthodromus* [2,4,5]. The buttonquails (Turnicidae) clustered with and were sisters to all other members of Lari. Turnicidae was positioned on a long branch, consistent with previous findings from studies, including those based on nuclear DNA sequences [8,46]. The underlying reasons for this long branch remain unclear.

## 4. Conclusions

The mitogenomes of six individuals of *Charadrius dubius* and one *Pluvialis fulva* from various locations across China were sequenced in this study. We also annotated and comprehensively described their mitogenomic characteristics. Importantly, this is the first intraspecies mitogenome analysis of *C. dubius*. Our study supports *Charadrius*’s monophyly, and *P. fulva* is nested within Charadriidae. The monophyly of Charadrii, Charadriidae (including *Pluvialis*), Scolopaci, Lari, Larida and Jacanida was strongly supported, as was the recognition of *Saundersilarus* as a distinct genus from *Chroicocephalus*. Maximum likelihood (ML) and Bayesian inference (BI) analyses confirmed the clade of ((Recurvirostridae + Haematopodidae) + Charadriidae) within the suborder Charadrii. Our study contributes six complete mitogenomes of *C. dubius* and one of *P. fulv*a, enriching the genetic resources of these species. However, additional samples of other species within Charadrii dae are needed to further address unresolved phylogenetic issues.

## Figures and Tables

**Table 1:** Details of the *Charadrius dubius* and *Pluvialis fulva* mitochondrial genomes sequenced in this study (* indicates nestlings; gender could not be determined).

Species	Sample ID	Collection Date	Gender	Collection Site
*C. dubius*	5	4 June 2018	*	Hongjiannao Lake, Shenmu, Shaanxi, China
*C. dubius*	6	4 June 2018	*	Hongjiannao Lake, Shenmu, Shaanxi, China
*C. dubius*	39	5 July 2018	Female	Tangyu, Lantian, Shaanxi, China
*C. dubius*	40	5 July 2018	Male	Tangyu, Lantian, Shaanxi, China
*C. dubius*	146	11 June 2020	*	Yuyang River, Yulin, Shaanxi, China
*C. dubius*	147	11 June 2020	*	Yuyang River, Yulin, Shaanxi, China
*P. fulva*	150	2 March 2020	Male	Xi’an Xianyang International Airport, Xi’an, Shaanxi, China

**Table 2:** Length and GC content of different areas in six individuals of *Charadrius dubius* and one *Pluvialis fulva.*

Speices	Sample ID	Length (bp)				GC Content (%)			
		Mitogenome	Of Protein-Coding Genes	rRNA	tRNA	Mitogenome	Of Protein-Coding Genes	rRNA	tRNA
*C. dubius*	5	16,866	11,418	2567	1553	44.6	45.3	45.6	41.7
	6	16,888	11,418	2538	1553	44.6	45.4	45.6	41.6
	39	16,911	11,400	2566	1556	44.6	45.3	45.7	41.5
	40	16,833	11,397	2568	1552	44.6	45.3	45.7	41.6
	146	16,926	11,400	2544	1553	44.5	45.3	45.5	41.6
	147	16,911	11,397	2544	1553	44.6	45.3	45.5	41.7
*P. fulva*	150	16,859	11,391	2539	1551	45.2	46.2	44.8	41.3

**Table 3:** GC skew and GC content in the mitogenomes of 10 Charadriidae species.

Species	AT Skew	GC Skew	AT%	GC%
*Charadrius alexandrinus*	0.14	−0.39	55.24	44.76
*Charadrius dubius*	0.14	−0.39	54.99	45.01
*Charadrius dubius* (No. 146)	0.14	−0.38	55.48	44.52
*Charadrius dubius* (No. 147)	0.14	−0.39	55.41	44.59
*Charadrius dubius* (No. 39)	0.14	−0.38	55.38	44.62
*Charadrius dubius* (No. 40)	0.14	−0.38	55.37	44.63
*Charadrius dubius* (No. 5)	0.14	−0.38	55.43	44.57
*Charadrius dubius* (No. 6)	0.14	−0.38	55.34	44.66
*Charadrius leschenaultii*	0.14	−0.39	55.47	44.53
*Charadrius mongolus*	0.13	−0.39	55.38	44.62
*Charadrius vociferus*	0.14	−0.40	55.58	44.42
*Pluvialis apricaria*	0.15	−0.40	54.37	45.63
*Pluvialis fulva*	0.15	−0.39	54.87	45.13
*Pluvialis fulva* (No. 150)	0.14	−0.39	54.76	45.24
*Pluvialis squatarola*	0.14	−0.38	54.27	45.73
*Vanellus cinereus*	0.15	−0.39	55.15	44.85
*Vanellus vanellus*	0.13	−0.38	55.47	44.53

## Data Availability

The original contributions presented in this study are included in the article. Further inquiries can be directed to the corresponding author.

## Supplementary Materials

The following supporting information can be downloaded at: https://www.mdpi.com/article/10.3390/genes15121642/s1, Figure S1: The ML tree of 114 Charadriiformes sequences based on 13 mitochondrial CDSs; Table S1: Taxonomic information and GenBank accession numbers for taxa used in the phylogenetic analysis; Table S2: Gene lengths and start/stop codon information for the 13 CDSs of *Charadrius dubius* and *Pluvialis fulva*.
