# Supplementary material for: Complete Mitochondrial Genomes of Pluvialis fulva and Charadrius dubius with Phylogenetic Analysis of Charadriiformes
Source: Genes (Basel). 2024 Dec 21;15(12):1642. doi: 10.3390/genes15121642 (PMC11675994; doi:10.3390/genes15121642)
Supplement: Supplementary file 1 [file genes-15-01642-s001.zip › Figure S1.pdf]

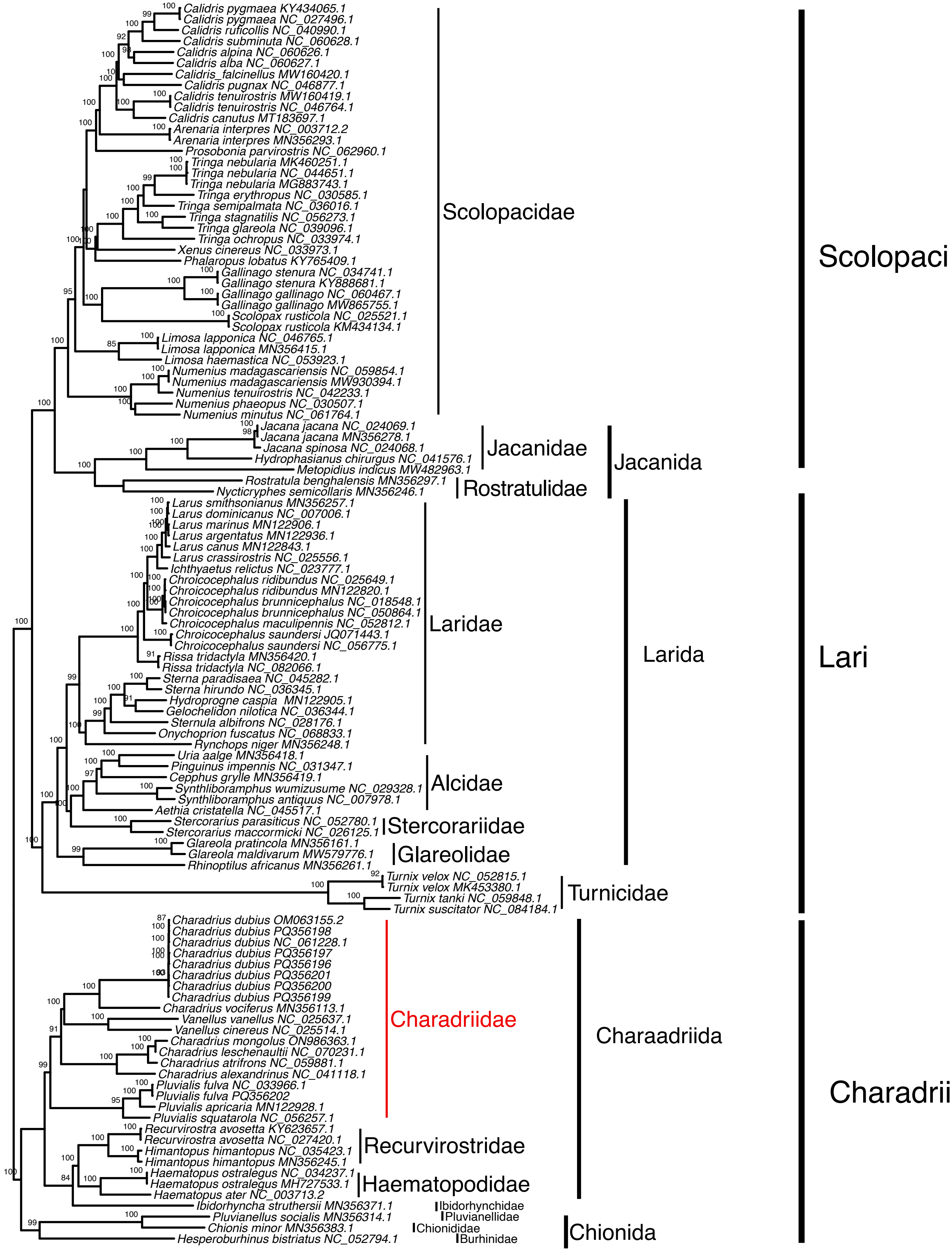

Calidris pygmaea KY434065.1
Calidris pygmaea NC_027496.1
Calidris ruficollis NC_040990.1
Calidris subminuta NC_060628.1
Calidris alpina NC_060626.1
Calidris alba NC_060627.1
Calidris_falcinellus MW160420.1
Calidris pugnax NC_046877.1
Calidris tenuirostris MW160419.1
Calidris tenuirostris NC_046764.1
Calidris canutus MT183697.1
Arenaria interpres NC_003712.2
Arenaria interpres MN356293.1
Prosobonia parvirostris NC_062960.1
Tringa nebularia MK460251.1
Tringa nebularia NC_044651.1
Tringa nebularia MG883743.1
Tringa erythropus NC_030585.1
Tringa semipalmata NC_036016.1
Tringa stagnatilis NC_056273.1
Tringa glareola NC_039096.1
Tringa ochropus NC_033974.1
Xenus cinereus NC_033973.1
Phalaropus lobatus KY765409.1
Gallinago stenura NC_034741.1
Gallinago stenura KY888681.1
Gallinago gallinago NC_060467.1
Gallinago gallinago MW865755.1
Scolopax rusticola NC_025521.1
Scolopax rusticola KM434134.1
Limosa lapponica NC_046765.1
Limosa lapponica MN356415.1
Limosa haemastica NC_053923.1
Numenius madagascariensis NC_059854.1
Numenius madagascariensis MW930394.1
Numenius tenuirostris NC_042233.1
Numenius phaeopus NC_030507.1
Numenius minutus NC_061764.1
Scolopacidae
Jacana jacana NC_024069.1
Jacana jacana MN356278.1
Jacana spinosa NC_024068.1
Hydrophasianus chirurgus NC_041576.1
Metopidius indicus MW482963.1
Jacanidae
Rostratula benghalensis MN356297.1
Nycticryphes semicollaris MN356246.1
Rostratulidae
Jacanida
Scolopaci
Larus smithsonianus MN356257.1
Larus dominicanus NC_007006.1
Larus marinus MN122906.1
Larus argentatus MN122936.1
Larus canus MN122843.1
Larus crassirostris NC_025556.1
Ichthyaetus relictus NC_023777.1
Chroicocephalus ridibundus NC_025649.1
Chroicocephalus ridibundus MN122820.1
Chroicocephalus brunnicephalus NC_018548.1
Chroicocephalus brunnicephalus NC_050864.1
Chroicocephalus maculipennis NC_052812.1
Chroicocephalus saundersi JQ071443.1
Chroicocephalus saundersi NC_056775.1
Rissa tridactyla MN356420.1
Rissa tridactyla NC_082066.1
Sterna paradisaea NC_045282.1
Sterna hirundo NC_036345.1
Hydroprogne caspia MN122905.1
Gelochelidon nilotica NC_036344.1
Sternula albifrons NC_028176.1
Onychoprion fuscatus NC_068833.1
Rynchops niger MN356248.1
Laridae
Uria aalge MN356418.1
Pinguinus impennis NC_031347.1
Cepphus grylle MN356419.1
Synthliboramphus wumizusume NC_029328.1
Synthliboramphus antiquus NC_007978.1
Aethia cristatella NC_045517.1
Alcidae
Stercorarius parasiticus NC_052780.1
Stercorarius maccormicki NC_026125.1
Stercorariidae
Glareola pratincola MN356161.1
Glareola maldivarum MW579776.1
Rhinoptilus africanus MN356261.1
Glareolidae
Larida
Turnix velox NC_052815.1
Turnix velox MK453380.1
Turnix tanki NC_059848.1
Turnix suscitator NC_084184.1
Turnicidae
Lari
Charadrius dubius OM063155.2
Charadrius dubius PQ356198
Charadrius dubius NC_061228.1
Charadrius dubius PQ356197
Charadrius dubius PQ356196
Charadrius dubius PQ356201
Charadrius dubius PQ356200
Charadrius dubius PQ356199
Charadrius vociferus MN356113.1
Vanellus vanellus NC_025637.1
Vanellus cinereus NC_025514.1
Charadrius mongolus ON986363.1
Charadrius leschenaultii NC_070231.1
Charadrius atrifrons NC_059881.1
Charadrius alexandrinus NC_041118.1
Pluvialis fulva NC_033966.1
Pluvialis fulva PQ356202
Pluvialis apricaria MN122928.1
Pluvialis squatarola NC_056257.1
Charadriidae
Recurvirostra avosetta KY623657.1
Recurvirostra avosetta NC_027420.1
Himantopus himantopus NC_035423.1
Himantopus himantopus MN356245.1
Recurvirostridae
Haematopus ostralegus NC_034237.1
Haematopus ostralegus MH727533.1
Haematopus ater NC_003713.2
Haematopodidae
Ibidorhyncha struthersii MN356371.1
Ibidorhynchidae
Pluvianellus socialis MN356314.1
Pluvianellidae
Chionis minor MN356383.1
Chionididae
Hesperoburhinus bistriatus NC_052794.1
Burhinidae
Charaadriida
Chionida
Charadrii
